# Dermoid Cyst of the Pancreas: A Report of an Unusual Case and a Review of the Literature

**DOI:** 10.1155/2013/375193

**Published:** 2013-11-14

**Authors:** Aynur Albayrak, Umran Yildirim, Metin Aydin

**Affiliations:** ^1^Diskapi Yildirim Beyazit Education and Research Hospital, Department of Pathology, 06110 Ankara, Turkey; ^2^Turgut Özal University of Medical Faculty, Department of Pathology, 06510 Ankara, Turkey; ^3^Duzce University of Medical Faculty, Department of General Surgery, 81650 Duzce, Turkey

## Abstract

Pancreatic dermoid cysts are a rare entity. Preoperative diagnosis is difficult. The diagnosis is generally taking intraoperative. A 20-year-old female presented with epigastric pain without nausea, vomiting, diarrhea, fever, jaundice, and weight loss of one-month duration. Ultrasonography and computed tomography showed a smooth borders, solid, hyperechoic tumor within midline abdomen, without any connection to the stomach or spleen. At surgery, the entire mass was excised off of the head and inferior part of pancreas. Histopathologic evaluation revealed the rare diagnosis of a dermoid cyst. The diagnosis is difficult preoperatively in evaluating cystic pancreatic lesions by imaging. Therefore, we want to summarize the literature on this rare entity knowledge.

## 1. Introduction

Dermoid cysts are congenital developmental abnormalities of germ cell origin derived from any of the three germinal layers, that is, ectoderm, entoderm, and mesoderm. Teratomas usually occur along the midline of the body, which is the route of germ cell migration during embryogenesis. These migrating germ cells may become “misplaced” en route to their appropriate organs, leading to the development of tumors later in life. The pancreas is extremely rare as a primary site. As true cysts, dermoid cysts are usually benign, well differentiated lesions. Pancreatic teratomas probably originate from aberrant germ cells arrested in migration to the gonads early in embryonic life. They are composed of tissue derived from any of the three germinal layers and may produce a wide variety of structures with different degrees of differentiation, including hair, teeth, cartilage, bone, and dermal appendages, such as hair follicles, sweat glands, and sebaceous material [1, 2]. They are an unusual entity with only 30 cases, to our knowledge, described in the world literature. We report the 31st case of a pancreatic cystic teratoma.

## 2. Case-Report

A case of a 20-year-old woman was admitted to our hospital due to vague epigastric pain without nausea, vomiting, diarrhea, fever, jaundice, and weight loss of one-month duration. The patient had no significant medical history. A physical examination demonstrated mild tenderness in the epigastrium but no evidence of an acute abdomen. Laboratory studies including functional tests of the pancreas, and the liver and kidneys showed normal values. The levels of serum carbohydrate antigenic determinant (CA19-9) and carcinoembryonic antigen (CEA) were not elevated. 

**Figure 1:**
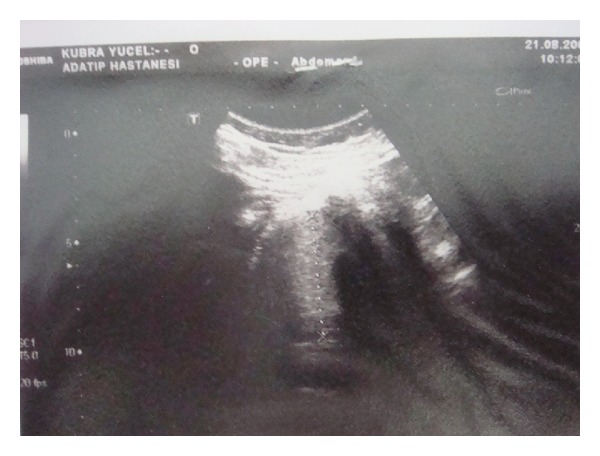
(Ultrasonographic (USG) examination): the lesion well-defined, solid, hyperechoic mass of pancreatic head-body, measuring 5 × 5 cm. No infiltration into the intra-abdominal organs is seen.

A CT scan revealed a lobular contoured solid mass with a 13 cm long axis, 6 cm transverse diameter, and 5 cm anterior to posterior diameter, localized between the head and inferior part of the pancreas, compressing the pancreas and surrounding tissues. Low density was dominant, but the lesion also included patchy high dense regions and an approximately 2 × 5 cm sized calcification. Ultrasonographic (USG) examination reveals that solid and hyperechoic mass lesion was observed in the midline of the abdomen. This mass is approximately 5 × 5 cm in size with smooth borders and does not invade the intra-abdominal organs (Figure 1). No definite adenopathy was noted at the celiac axis origin or in any peripancreatic area. 

At surgery, the entire mass was excised off of the head and inferior part of pancreas and sent for histopathologic examinations.

**Figure 2:**
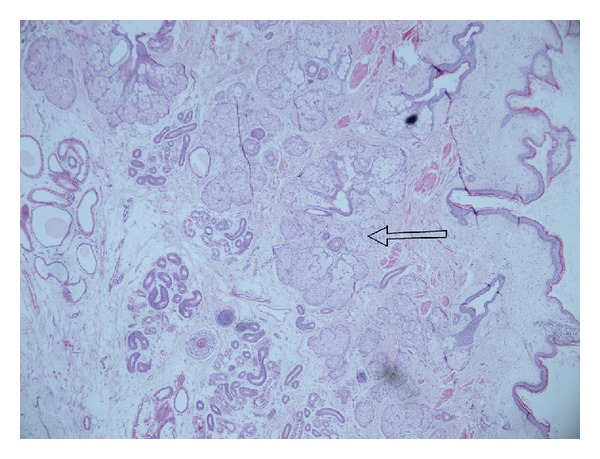
Microscopic examination showed mature squamous epithelium with formation of a granular cell layer with underlying sebaceous glands (arrow), filling the cyst with laminated keratin (hematoxylin and eosin (H&E), 100x magnification).

Macroscopically, a gray-brown colored, 10 × 7 × 5 cm sized mass with irregular surface was found. There was a glazing compound and a 9 cm sized hair ball on the upper surface of this mass. The cross-section was yellow-white in color. In some regions, there was cystic lumination (multiloculation). The different parts of the lesion were observed in seven cassettes. On histological examination, it was revealed that the cyst wall was lined by mature stratified squamous epithelium and surrounded by lymphoid tissue containing germinal centers and sebaceous glands. In the lumen of the cyst, masses of keratinous debris were detected (Figure 2).

The patient died from postoperative complication, that is, massive intra-abdominal hemorrhage and hypovolemic shock.

## 3. Discussion

Teratomas are widely believed to arise from embryonic inclusions of skin at the time of neural groove closure, hence their characteristic midline localization. Two subtypes of teratomas have been described: mature and immature. Mature teratomas are further classified as either solid or cystic, with the latter also known as “dermoid cyst.” Although dermoid cysts most commonly develop within the ovaries, they have been shown to occur anywhere along the route of ectodermal cell migration, usually in the midline. Localizations in the testis, cranium, brain, mediastinum, omentum, retroperitoneum, and sacrococcygeal region have all been described. Pancreatic dermoid cysts are extremely rare [2, 3].

Following the description of a case of a mature cystic teratoma (MCT) of the pancreas by Kerr in 1918 [3, 4], the characteristics of these tumors have been identified in several case studies. Pancreatic MCTs usually develop at a young age, with a mean age at diagnosis of 34.7 years (range 2–74 years). There is also a slight male preponderance in reported cases (59% men, 41% women) [3, 4]. Although such teratomas have been shown to develop anywhere in the pancreas, most lesions are located in the body or the head (47% and 41%, resp.), while the tail of the pancreas is less frequently involved (12%) [4, 5]. In our patient, the lesion was situated on the inferior portion of the pancreatic head. A comparison of our case with other reported cases in the literature is provided in Table 1. 

While most patients with pancreatic MCTs are asymptomatic, lesions are usually discovered incidentally. Patients may complain of nonspecific gastrointestinal symptoms such as nausea and vomiting, weight loss, anorexia, fatigue, abdominal pain, back pain, and fever [3–5]. Laboratory tests are usually unremarkable unless the lesion hinders the flow of biliary or pancreatic fluids. Serum levels of CEA and CA19-9, which have traditionally been used for the evaluation of cystic neoplasms of the pancreas, are considerably lower in dermoid cysts [2, 3], although this was not the case in our patient. 

The radiologic appearance of pancreatic dermoid cysts, as with other MCTs, depends on their composition. A combination of radiologically detectable fat, fat-fluid levels, and calcium within the same lesion is considered to be highly suggestive of a mature teratoma [6, 7]. On ultrasonographic examination, an MCT may appear as a well-defined cystic mass with distinct margins but without septations. Areas of high fat content appear hyperechoic, whereas calcified tissues are seen as focal areas of high intensity with acoustic shadowing [2, 4, 7]. 

On computerized tomography scans, an MCT of the retroperitoneum manifests as a complex mass containing a well-circumscribed fluid component, adipose tissue, and calcifications. The presence of hypoattenuated fat areas within the cyst is considered highly suggestive of cystic teratoma, particularly if calcifications are detected in the cyst wall [8]. 

On further evaluation with magnetic resonance imaging (MRI), T1-weighted images would show cystic lesions with distinct margins and fat-fluid levels of low intensity. Findings on both CT and MRI are highly specific, providing an accurate diagnosis in most cases [2, 4, 7].

A definite diagnosis needs to be made by excisional biopsy or fine needle aspiration cytology (FNAC). In 1991, Markovsky and Russin [9] described a case of a patient preoperatively diagnosed with cystic teratoma by FNAC. Authors reported on the presence of mature benign squamous cells, keratin debris, and inflammatory cells on cytological examination, which they considered diagnostic citing that other lesions of the pancreas such as pseudocysts, pancreatitis, and degenerated carcinomas would be expected to lack specific histological elements [9]. The current recommendation is to perform FNAC to confirm a preoperative diagnosis if radiological evidence is inconclusive for the evaluation of a cystic lesion of the pancreas [2]. Koomalsingh et al. reiterated that FNAC should be reserved to asymptomatic patients and individuals considered at high risk for surgery [2]. Despite the available modalities, a preoperative diagnosis of a pancreatic cystic lesions may be elusive, particularly for larger lesions (>3 cm). In a study by Volmar et al., the preoperative and postoperative diagnoses of 1000 patients with cystic lesions of the pancreas were compared. Investigators reported on a false positive rate of 0.3% and a false negative rate of 14.3% following preoperative evaluation with US, CT, or EUS guided FNAC [10]. 

Dermoid cysts of the pancreas are true cysts. The cyst wall, which surrounds the lesion, is lined by a single layer of keratinizing stratified squamous epithelium, and the underlying connective tissue may contain adnexal tissue, sebaceous glands, lymphoid tissue, and even inflammatory cells. Dermoid cysts often contain thick, pasty, doughy sebaceous secretions. Fully differentiated tissues from one or more germ cell layers, most commonly the ectoderm, including hair, teeth, calcium, cartilage, and dermal appendages, such as hair follicles, sweat glands, and sebaceous material, are also usually encountered [2–4, 11]. 

The differential diagnoses of pancreatic cystic lesions include pseudocyst, neoplastic cysts such as mucinous and serous cystadenoma, intraductal papillary mucinous neoplasm (IPMN), and solid pseudopapillary tumor. Although US, CT, and MRI are undoubtedly helpful, findings are far from being pathognomonic [12, 13]. As opposed to true cysts, pseudocysts lack a true epithelial lining. Mucinous cystic neoplasms, on the other hand, are true cystic neoplasms lined by mucinous epithelium that develop separate from the ductal system. They often contain a cellular subepithelial stroma which may resemble ovarian stroma [14]. Serous cystadenomas, which can be either microcystic or macrocystic, are characterized by the presence of serous, cuboidal, clear cells with uniform, round hyperchromatic nuclei [15]. A cystic lesion with a mucinous epithelium lining that is connected to the native ducts is classified as IPMN [16]. Solid pseudopapillary tumors are a distinctive subtype of pancreatic lesions that are histopathologically characterized by the presence of degenerative pseudopapillae, loosely cohesive cells with grooved nuclei, and aggregates of large hyaline globules [17]. Lymphoepithelial cysts, which are also most commonly located in the pancreas (tail and body, followed by the head and neck), resemble dermoid cysts and may be distinguished by the absence of epidermal appendages such as hair follicles or sebaceous glands, which are more frequently associated with the latter [18]. Furthermore, mucinous epithelium, respiratory-type mucosa, sebaceous units, and hair follicles are more readily identifiable in dermoid cysts rather than in lymphoepithelial or epidermoid cysts. Another distinguishing feature is that suppurative infections occur more frequently in dermoid cysts than in other “squamous lined” pancreatic cysts [1, 3, 19, 20]. 

Although malignant degeneration of pancreatic dermoid cysts has yet to be described, it is essential that histopathological evaluation includes complete sampling of the cyst wall in order to exclude the presence of immature foci (most commonly neuroepithelial type), since 7–10% of other retroperitoneal teratomas have been reported to be malignant [21]. 

Despite their benign nature, ambiguity regarding a diagnosis often leads to surgical resection, which remains the gold standard treatment for MCTs. In a recent review, it was established that more than 70% of excised pancreatic cystic lesions reported were either malignant or premalignant, and based on these findings it is recommended that surgical excision be undertaken for any symptomatic cystic lesion as well as any lesion larger than 2-3 cm in size, particularly if discovered in an elderly patient [5]. Not only would excision of the lesion provide definitive diagnosis, it is also expected to lessen symptoms [1, 5]. External drainage should be avoided as it may be associated with a risk of developing a persistent fistula requiring surgical intervention. Simple excision (enucleation or cystectomy) of the lesion, preceded by intraoperative frozen section, usually suffices as recurrence or evidence of malignant degeneration, has not been reported [4, 5]. 

Intraoperative frozen examination is a useful tool that may help prevent overtreatment of pancreatic masses in young individuals.

## Figures and Tables

**Table 1:** A presentation of the cases of dermoid cyst of the pancreas found in the literature.

Author	Year	Age	Sex	Size	Localization	Symptoms	Treatment
Kerr AA	1918	55	F	—	Head/body	Gastritis? Dyspepsia?	Marsupialization+ (after 2 months) resection
Dennis WA	1923	33	F	—	Head	Pain and palpable mass	Marsupialization
Decourcy JL	1943	2	F	—	Body	Emesis, vomiting	Enucleation
Iovchec II	1972	8	M	—	Body	Emesis and pain	Drainage
Pomosov DV	1973	6	M	—	Tail	Pain	SPG
Assawamatiyanont S	1977	11	F	9 × 8 × 6	Body	Abdominal mass	Enucleation
Lazaro DaSilva A	1984	21	M	—	Body	Painless mass and emesis, constipation	Resection after drainage
Vermeulen	1990	46	M	—	Tail	İncidental	SPG
Mester M	1990	25	F	8 × 8 × 8	Head	Abdominal pain	Enucleation
Jacobs JE	1993	57	F	5.9 × 6.5 × 6.6	Body	pain and weight losing	Enucleation
Markovsky V	1993	53	F	20 × 20 × 11	Body	Abdominal pain	Enucleation
Lacono C	1993	26	F	12 × 12 × 12	Head	Pain and fever	DPC
Das PC	1996	4 months	F	9.5 × 8.5	Body-tail	Abdominal mass at palpation	Enucleation
Lushpai VP	1997	—	—	—	—	—	—
Fernandez-Cebrian JM	1998	74	M	10 × 8 × 9	Body-tail	Backache pain	SPG
Strasser G	2002	44	M	7 × 5 × 7	Head	Pain	—
Salimi J	2004	21	M	—	Head	Jaundice and weight loss	Enucleation
Seki M	2005	60	F	22 × 19 × 14	Body	İncidental	Enucleation
Seki M	2005	57	M	5.5 × 3.7 × 3.3	Body	İncidental	PM
Koomalsingh KJ	2006	52	M	3.5 × 3 × 2.2	Tail	Pain	SPG
Rıvkıne E	2007	45	F	2.8 × 1.8 × 2.7	Head	Abdominal pain	Resection
Tucci G	2007	64	M	8.5 × 3.0	Tail	İncidental	Pancreatectomy
Yoon WJ	2008	57	M	—	Head	Abdominal pain	—
Zhang AY	2008	67	M	4.6 × 3 × 2.2	Body	İncidental	Distal pancreatectomy and splenectomy
Scheele J	2010	40	M	6.4 × 4.9 × 3.8	Head-body	Upper abdominal pain	Partial duodenopancreatectomy
Badıa AC	2010	43	F	15 × 10 × 10	Body	Epigastric pain and emesis	Partial cystectomy
Ameur B	2011	64	M	10 × 8	Head	Epigastric pain	Partial cystectomy
Urata T	2012	58	M	—	Tail	Epigastric and dorsal pain	—
Degrate	2012	61	M	—	Uncinate	Asymptomatic	DPC
Lane	2012	63	M	5.7 × 5.1 × 4.1	Body	Lower abdominal pain	Resection
Albayrak A	2013	20	F	10 × 7 × 5	Head-body	Epigastric pain	Enucleation

SPG: splenopancreatectomic gauche, DPC: duedunopancreatectomy cephalic, PM: pancreatectomy mediane, —: nonspecific.
